# Impact of Long COVID on health and quality of life

**DOI:** 10.12688/hrbopenres.13516.1

**Published:** 2022-04-22

**Authors:** Liam O’ Mahony, Tanja Buwalda, Matthew Blair, Brian Forde, Nonhlanhla Lunjani, Anoop Ambikan, Ujjwal Neogi, Peter Barrett, Eoin Geary, Nuala O'Connor, Jennifer Dineen, Gerard Clarke, Eric Kelleher, Mary Horgan, Arthur Jackson, Corinna Sadlier

**Affiliations:** 1 Department of Medicine, University College Cork, Cork, Ireland; 2 School of Microbiology, University College Cork, National University of Ireland, Cork, Ireland; 3 APC Microbiome Ireland, University College Cork, National University of Ireland, Cork, Ireland; 4 Long COVID Ireland Support Group, Cork, Ireland; 5 Department of Infectious Disease, Cork University Hospital, Cork, Ireland; 6 The Systems Virology Lab,Division of Clinical Microbiology, Department of Laboratory Medicine, Karolinska Institute, ANA Futura, Campus Flemingsberg, Stockholm, Sweden; 7 Department of Public Health, HSE South, St Finbarr’s Hospital, Cork, Ireland; 8 Liaison Psychiatry Service, Cork University Hospital, Cork, Ireland; 9 Department of Psychiatry and Neurobehavioural Science, University College Cork, National University of Ireland, Cork, Ireland; 10 Irish College of General Practitioners, ICGP, Dublin, Ireland; 11 Department of Neurophysiology, Cork University Hospital, Cork, Ireland

**Keywords:** Long COVID, PASC, COVID-19

## Abstract

**Background**: The aim of this study was to measure the impact of post-acute sequelae of COVID-19 (PASC) on quality of life, mental health, ability to work and return to baseline health in an Irish cohort.

**Methods**: We invited individuals with symptoms of COVID-19 lasting more than 14 days to participate in an anonymous online questionnaire. Basic demographic data and self-reported symptoms were recorded. Internationally validated instruments including the patient health questionnaire somatic, anxiety and depressive symptom scales (PHQ-SADS), the Patient Health Questionnaire-15 (PHQ-15) and Chadler fatigue scale (CFQ) were used.

**Results**: We analysed responses from 988 participants with self-reported confirmed (diagnostic/antibody positive; 81%) or suspected (diagnostic/antibody negative or untested; 9%) COVID-19. The majority of respondents were female (88%), white (98%), with a median age of 43.0 (range 15 – 88 years old) and a median BMI of 26.0 (range 16 – 60). At the time of completing this survey, 89% of respondents reported that they have not returned to their pre-COVID-19 level of health. The median number of symptoms reported was 8 (range 0 to 33 symptoms), with a median duration of 12 months (range 1 to 20 months) since time of acute infection. A high proportion of PASC patients reported that they have a moderate or severe limitation in their ability to carry out their usual activities, 38% report their ability to work is severely limited and 33% report a moderate, or higher, level of anxiety or depression.

**Conclusion**: The results of this survey of an Irish cohort with PASC are in line with reports from other settings, and we confirm that patients with PASC reported prolonged, multi-system symptoms which can significantly impact quality of life, affect ability to work and cause significant disability. Dedicated multidisciplinary, cross specialty supports are required to improve outcomes of this patient group.

## Introduction

Severe acute respiratory syndrome coronavirus 2 (SARS-CoV- 2) causes a broad spectrum of manifestations ranging from asymptomatic infection to fatal coronavirus disease 2019 (COVID-19) ^1, 2^. Following acute infection, a proportion of individuals experience prolonged symptoms which can significantly impact daily function, quality of life and cause disability, termed long COVID, post-COVID condition or post-acute sequelae of COVID-19 (PASC) ^3^.

Symptoms of PASC are broad ranging and include fatigue, headache, sleep disturbance as well as specific organ system manifestations such as cardiopulmonary, neurocognitive, and symptoms of anxiety and depression. Multisystem involvement is common. Symptoms may be new onset following initial recovery from an acute COVID-19 episode or persist from the initial illness. They may also fluctuate or relapse over time ^4^.

PASC is reported to affect between 10 and 30% of mild-moderate community managed COVID-19 cases up to three months after infection ^5, 6^. Symptoms improve or resolve slowly over time for the majority of patients with PASC however persistence of symptoms for more than one year has been reported in large numbers of patients. The Office of National Statistics in the UK report that over 1.3 million people are suffering from long COVID with over 500,000 experiencing symptoms for more than 1 year. 

Currently no consensus diagnostic criteria exist for PASC.

The National Institute for Health and Care Excellence (NICE) defines long COVID as symptoms that continue or develop after acute COVID-19 infection which cannot be explained by an alternative diagnosis. This term includes ongoing symptomatic COVID-19, from four to 12 weeks post-infection, and post-COVID-19 syndrome, beyond 12 weeks post-infection.

The National Institutes of Health (NIH) use the US Centers for Disease Control and Prevention’s (CDC) definition of long COVID or post COVID condition, which describes the condition as sequelae that extend beyond four weeks after initial infection ^7^.

The World Health Organisation (WHO) developed a clinical case definition of post COVID-19 condition by Delphi methodology in October 2021. It describes post COVID-19 condition as occurring *“in individuals with a history of probable or confirmed SARS CoV-2 infection, usually three months from the onset of COVID-19 with symptoms that last for at least two months and cannot be explained by an alternative diagnosis. Common symptoms include fatigue, shortness of breath, cognitive dysfunction but also others and generally have an impact on everyday functioning. Symptoms may be new onset following initial recovery from an acute COVID-19 episode or persist from the initial illness. Symptoms may also fluctuate or relapse over time”.*

To-date research efforts have focused on the acute phase of COVID-19 and development of a vaccine. The number of studies investigating the immunobiology and treatment of PASC are limited. Suggested mechanisms underlying symptoms of PASC include persistent virus or viral antigens in tissues causing inflammation, triggering of autoimmunity, neuroinflammation, endothelial dysfunction, microvascular thrombosis and dysbiosis or the microbiome and virome ^7– 11^.

We previously demonstrated that levels of multiple cytokines, including T _H_2 cytokines such as interleukin (IL)-4, macrophage-derived chemokine (MDC) and thymic stromal lymphopoietin (TSLP), remained elevated for an extended period of time following SARS-CoV-2 infection ^12^. We also identified changes in metabolism that associate with PASC including low levels of circulating tryptophan and serotonin ^13^.

Many of the symptoms reported with PASC overlap with those of dysautonomia ^14^. Post-viral dysautonomia is a well-described entity ^15^. The autonomic nervous system is crucial in any host immune response through the vagal anti-inflammatory reflex ^16^. PASC may represent a “fall-out” of the autonomic response to the initial COVID-19 infection. This has been described in the literature both in case reports and case series ^17, 18^. Postulated mechanisms include autoantibody formation against ganglionic acetylcholine receptors and angiotensin receptors, direct invasion of the brainstem by COVID-19 altering medullary function, direct effect on the extracardiac post-ganglionic sympathetic nerves as well as cardiovascular deconditioning ^19– 22^.

It is probable that multiple mechanisms contribute to PASC symptoms.

To date no clear biochemical or radiological features exist to confirm a diagnosis of PASC and there are potentially several clinical phenotypes with different presentations, prognoses, and outcomes.

In addition there are no proven treatments or consensus rehabilitation guidance.

As millions worldwide continue to become infected with SARS-CoV-2, the public health implications of PASC and the need to understand and respond are increasingly pressing.

In Ireland, as of January 2022, the Health Protection Surveillance Center (HPSC) have reported more than 1 million cases of SARS-CoV-2 infection over 6000 deaths reported. There is no published data on prevalence of PASC in the Irish setting and as yet no structured resourced healthcare strategy.

The aim of this study was to measure the impact of PASC on quality of life, mental health, ability to work and return to baseline health within the Irish population.

## Methods

We conducted an online survey ^23^ of people over 18 years of age living in Ireland with suspected and confirmed COVID-19 from September 28, 2021 to January 24, 2022.

The survey was launched at a patient focused long COVID Webinar hosted by Alimentary Pharmabiotic Centre (APC) Microbiome Ireland and promoted by Irish COVID-19 support groups (e.g. long COVID Ireland Support group) and social media platforms (e.g. Twitter, Facebook, Instagram).

We analysed responses from 988 participants with self-reported confirmed (diagnostic/antibody positive; 81%) or suspected (diagnostic/antibody negative or untested; 9%) COVID-19 with symptoms lasting over 14 days. 

The study was approved on September 13th 2021 by the Clinical Research Ethics Committee of the Cork Teaching Hospitals (CREC) ECM 4 (f) 7.9.21. Google Forms software was used to create and distribute the online survey. All participants provided digital informed consent. Survey responses were anonymised and contained no personal identifiable information. No email or IP addresses were collected.

The survey consisted of 30 question stems, some with multiple choice options. Symptoms experienced by respondents during acute COVID-19 illness and persistent symptoms were grouped as systemic, respiratory, cardiovascular, gastrointestinal, neuropsychiatric or musculoskeletal.

Internationally validated instruments including the patient health questionnaire somatic, anxiety and depressive symptom scales (PHQ-SADS) ^24^, which incorporates the Patient Health Questionnaire-9 (PHQ-9) ^25^, the Generalized Anxiety Disorder 7-item scale (GAD-7) ^26^ and the Patient Health Questionnaire-15 (PHQ-15) ^24^ were used.

The PHQ-9 ^25^ utilises a continuous measure with scores from 0 to 27 and cut-off points of 5 (mild), 10 (moderate), 15 (moderately severe) and 20 (severe) representing levels of depressive symptoms. 

The GAD-7 ^26^ is a seven item anxiety scale which can be used to grade severity of Generalised Anxiety Disorder (GAD) symptoms. Scores can range from 0 to 27, with 5 (mild), 10 (moderate) and 15 (severe) representing levels of anxiety symptoms. The GAD-7 (four weeks) asks the first five items of the panic module. A positive response to the first panic question alone has a sensitivity and specificity of 93% and 78% respectively while each additional “yes” response to the remaining four items increases specificity with only a minimal decline in sensitivity ^27^.

The PHQ-15 ^24^ is a screening somatic symptom subscale which includes 15 somatic symptoms which account for more than 90% of physical symptoms seen in primary care. The PHQ-15 asks patients to rate how much they have been bothered by each symptom during the past month on a 0 (“not at all”) to 2 (“bothered a lot”) scale. Thus, the total score ranges from 0 to 30, with cut-off points of 5, 10 and 15 representing thresholds for mild, moderate and severe somatic symptom severity, respectively. 

The final item on the PHQ-SADS is the respondent's global rating of symptom-related difficulty and asks how difficult have these problems made it for you to do your work, take care of things at home, or get along with other people? ^28^

The Chalder Fatigue scale (CFQ) ^29^ is a questionnaire which measures the severity of tiredness in illness that can cause fatigue. Items are rated on a 4-point Likert scale (0 = better than usual, 1 = no more than usual, 2 = worse than usual, 3 = much worse than usual), with higher scores indicating greater fatigue. A CFQ Score (0–33) mean “Likert” score was 24.4 (SD 5.8) for chronic fatigue syndrome (CFS) sufferers and 14.2 (SD 4.6) for a community sample.

The EQ-5D-5L ^28^ measures generic health status. Each of the 5 dimensions comprising the EQ-5D descriptive system is divided into 5 levels of perceived problems, namely Level 1: indicating no problem; Level 2: indicating slight problems; Level 3: indicating moderate problems; Level 4: indicating severe problems and finally Level 5: indicating extreme problems. Participant ‘Imagined health today’ scores are a self-reported score between 0 and 100 of participants’ imagined health on the day of reporting.

Participants could take breaks during the survey and progress was saved for up to 30 days to allow respondents to return to the survey at a later time. Questions that mentioned technical terms included a description in plain language. Questions were not mandatory and participants were able to skip to the next one.

### Statistical analysis

Prediction models were generated using decision tree classification algorithm. R package rpart v4.1.15 with default settings was used for generating the model. The total data was first split into train (70%) and test data (30%) for model training and model prediction respectively. The R function sample.split from the package caTools v1.18.2 was used to split the data. Confusion matrix was calculated to access the model accuracy using confusionMatrix function from the package caret v6.0.90. Similarity distance based approach was used for the clustering. Firstly, Gower’s distance metric was computed using the R function daisy from the package cluster v2.1.2. The distance matrix was then used for the clustering using partitioning around medoids (PAM) algorithm. Number of clusters in the data was predicted by calculating silhouette width. Dimensionality reduction algorithm t-SNE was applied on the gower distance matrix and plotted on 2D space. R package Rtsne v0.15 was used for executing t-SNE algorithm.

## Results

### Baseline characteristics of the PASC Cohort

 A total of 988 respondents completed the online survey. Missing data were excluded from analysis. The majority of survey respondents were female (88%), white (98%), with a median age of 43.0 (range 15 – 88 years old) and a median body mass index (BMI) of 26.0 (range 16 – 60). Respondents from 31 counties were included, with the highest numbers of participants being from Dublin (24%), Cork (23%), Kildare (6%) and Galway (4%). The majority of participants had obtained a third level qualification (34% attained a postgraduate degree, 29% obtained a certificate or diploma, 23% possess a university degree).

**Figure 1.**
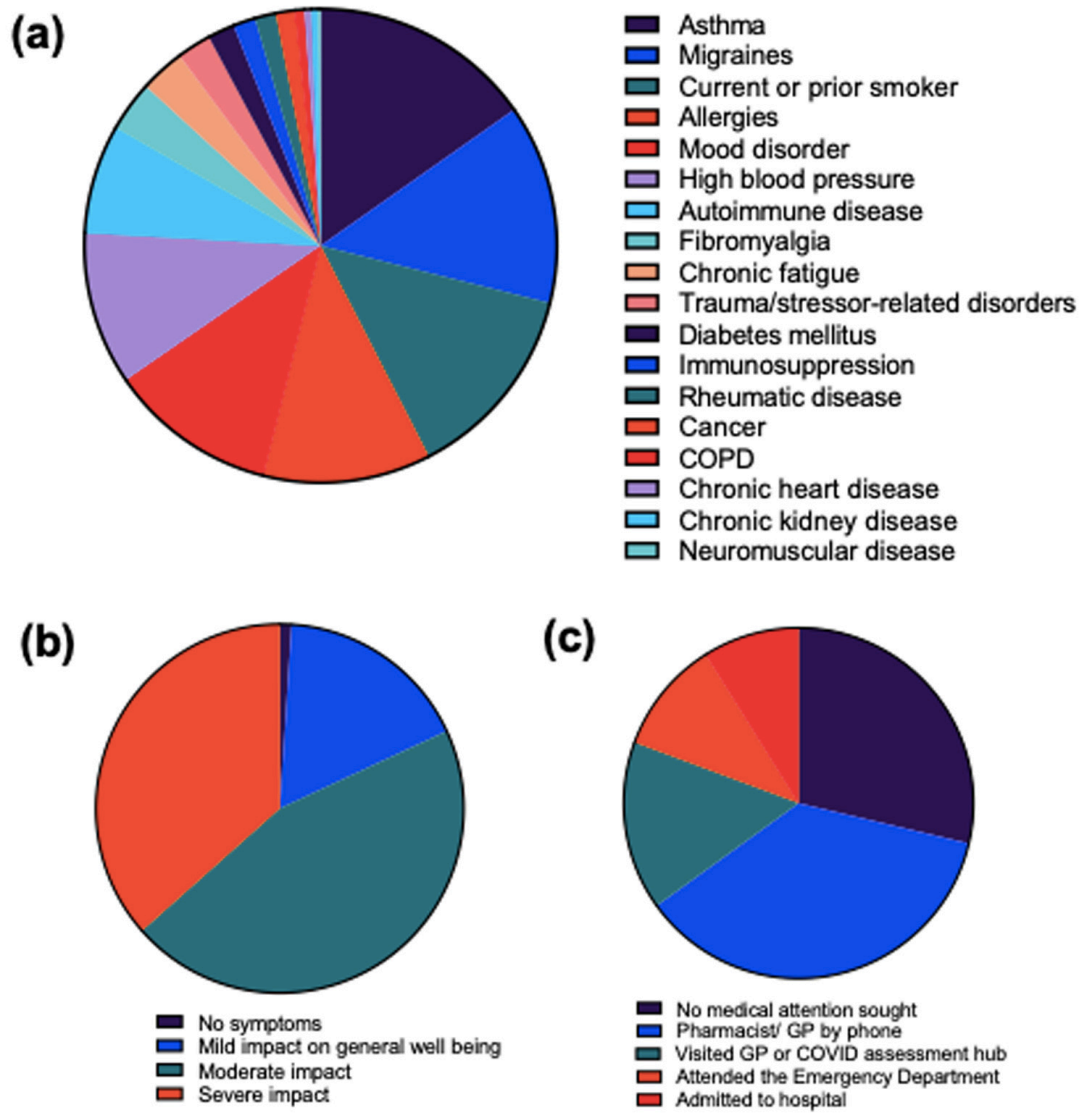
Baseline Characteristics. **a**. Pre-existing comorbidities. **b**. Self-assessment of acute COVID-19 infection severity. **c**. Level of care sought for acute COVID-19 infection.

Acute SARS-CoV-2 infection was confirmed by polymerase chain reaction (PCR) test for 81% of participants, infection in 3% of participants was confirmed by antigen test, while the remainder were suspected cases. The most common pre-existing comorbidities included asthma (16%), migraines (14%), mood disorders (12%) or allergies (12%) Figure 1a.

Patients’ self-assessment of acute infection severity ranged from mild impact on general well-being (17%), moderate impact (45%) to severe impact (37%), Figure 1b. Many patients did not seek medical advice (29%), or only sought advice by phone (36%), while 9% of participants were admitted to hospital during the acute phase of infection ( Figure 1c). Of those hospitalised, the median length of hospital stay was 4 days (range 1 to 149 days).

### Predominant symptoms during acute COVID-19 disease and PASC

**Figure 2.**
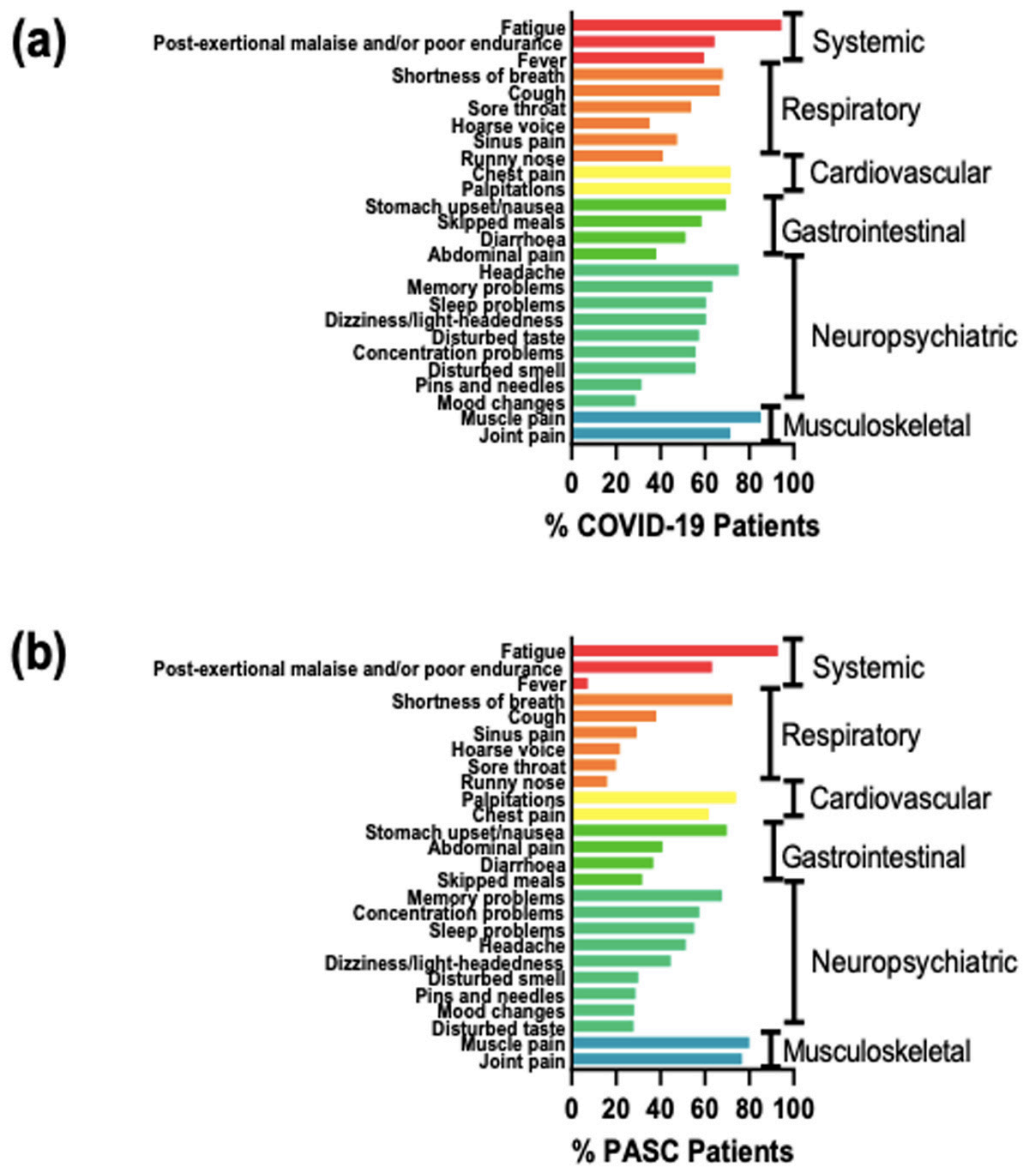
**a**. Acute COVID-19 Symptoms. **b**. PASC Symptoms.

 Symptoms reported were sub-grouped as systemic, respiratory, cardiovascular, gastrointestinal, neuropsychiatric or musculoskeletal. During acute COVID-19 disease, the majority of patients experienced many of these symptoms, with more than two thirds of patients experiencing fatigue, post-exertional malaise, shortness of breath, cough, chest pain, palpitations, stomach upset/nausea, headache, memory problems, muscle pain and joint pain ( Figure 2a). The median number of symptoms experienced during SARS-CoV-2 infection was 14, ranging from 0 to 34.

### Predominant symptoms during study participation

At the time of completing this survey, 89% of respondents reported that they have not returned to their pre-COVID-19 level of health. Certain symptoms, e.g. fever, decreased in prevalence amongst PASC patients ( Figure 2b). However, more than two thirds of PASC patients continue to experience fatigue, post-exertional malaise, palpitations, chest pain, stomach upset/nausea, memory problems, muscle pain and joint pain ( Figure 2b).

In addition, PASC patients reported new symptoms that were not present previously, which include tinnitus (38%), ear ache (31%), menstrual abnormalities (31%), mouth ulcers (28%), skin rash (27%), new allergies (16%) and sexual dysfunction (13%). The median number of symptoms that were being experienced at the time of survey completion was 8 (range 0 to 33 symptoms), with a median duration of 12 months (range 1 to 20 months) since time of acute infection.

27% of PASC patients felt that their symptoms were improving over time, while 43%, 23% and 7% of patients felt that symptoms are persistent, relapsing or are worsening over time, respectively. Overall, disease symptoms had a mild (24%), moderate (43%) or severe impact (33%) on general well-being.

### 5Q-5D-5L

**Figure 3.**
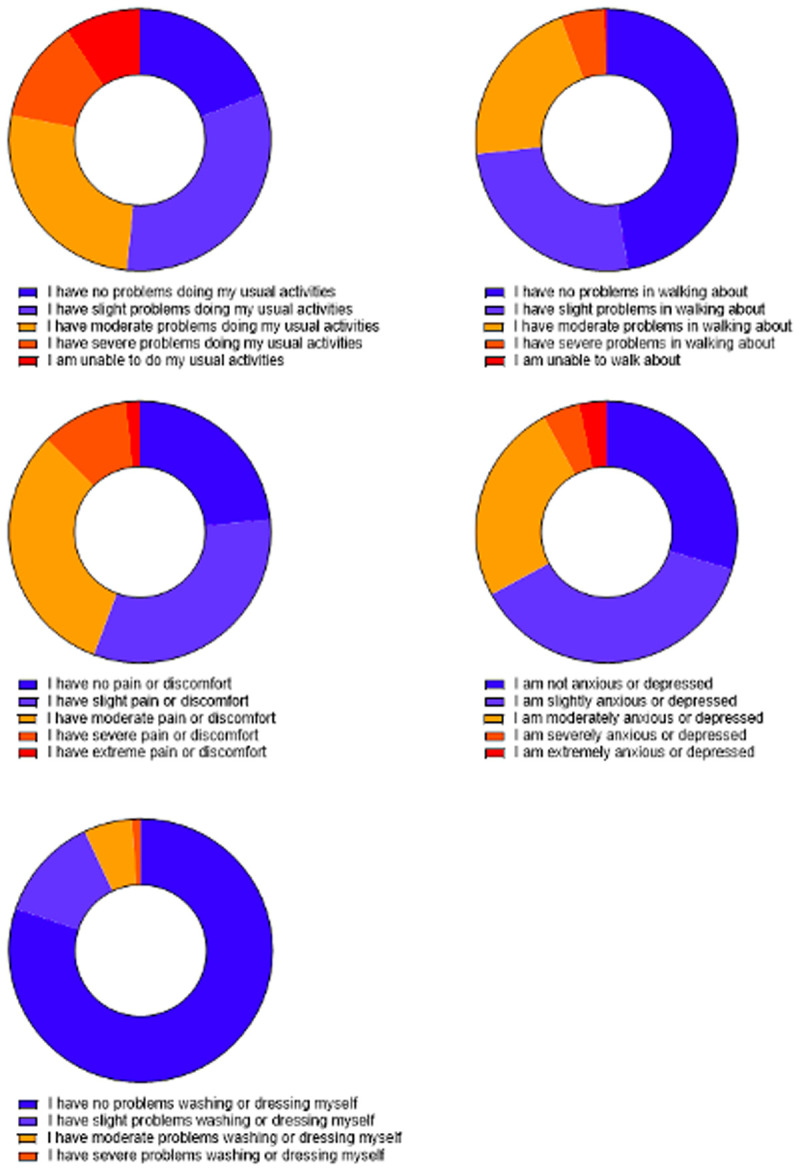
5Q-5D-5L measure of Health Status Today.

 When asked about their health status today (day of questionnaire), a high proportion of PASC patients indicate that they have a moderate or severe limitation in their ability to carry out their usual activities (48%), and in their mobility (27%), with 44% experiencing a moderate, or higher, level of pain and 33% report a moderate, or higher, level of anxiety or depression ( Figure 3).

**Figure 5.**
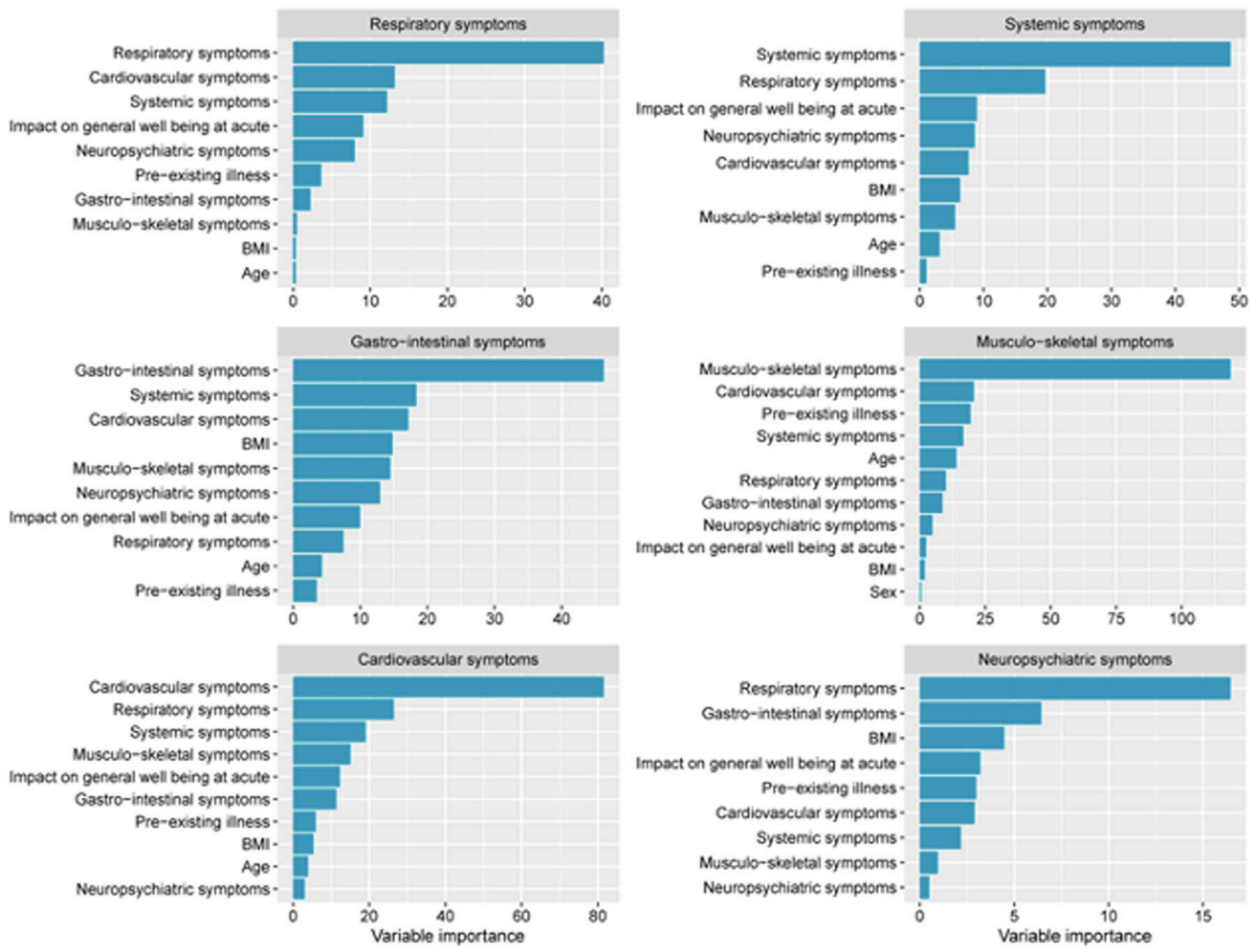
PASC symptom prediction models.

Using a classification algorithm decision tree, individual models were created for predicting patient's PASC symptoms based on their baseline characteristics and features associated with their acute phase of COVID-19 disease. The models with highest prediction accuracy were those predicting respiratory symptoms (63% accuracy) and musculoskeletal symptoms (61% accuracy), followed by cardiovascular symptoms (59% accuracy), gastrointestinal symptoms (59% accuracy), neuropsychiatric symptoms (54% accuracy) and systemic symptoms (49% accuracy). Variable importance assigned by the models showed that the presence of respiratory, systemic, gastrointestinal, musculoskeletal or cardiovascular PASC symptoms in these models were most heavily influenced by respiratory, systemic, gastrointestinal, musculoskeletal or cardiovascular symptoms respectively during acute disease ( Figure 5). However, PASC neuropsychiatric symptoms such as brain fog, sleep problems, and concentration problems were best predicted by respiratory symptoms and gastrointestinal symptoms during acute disease and BMI ( Figure 5).

**Figure 6.**
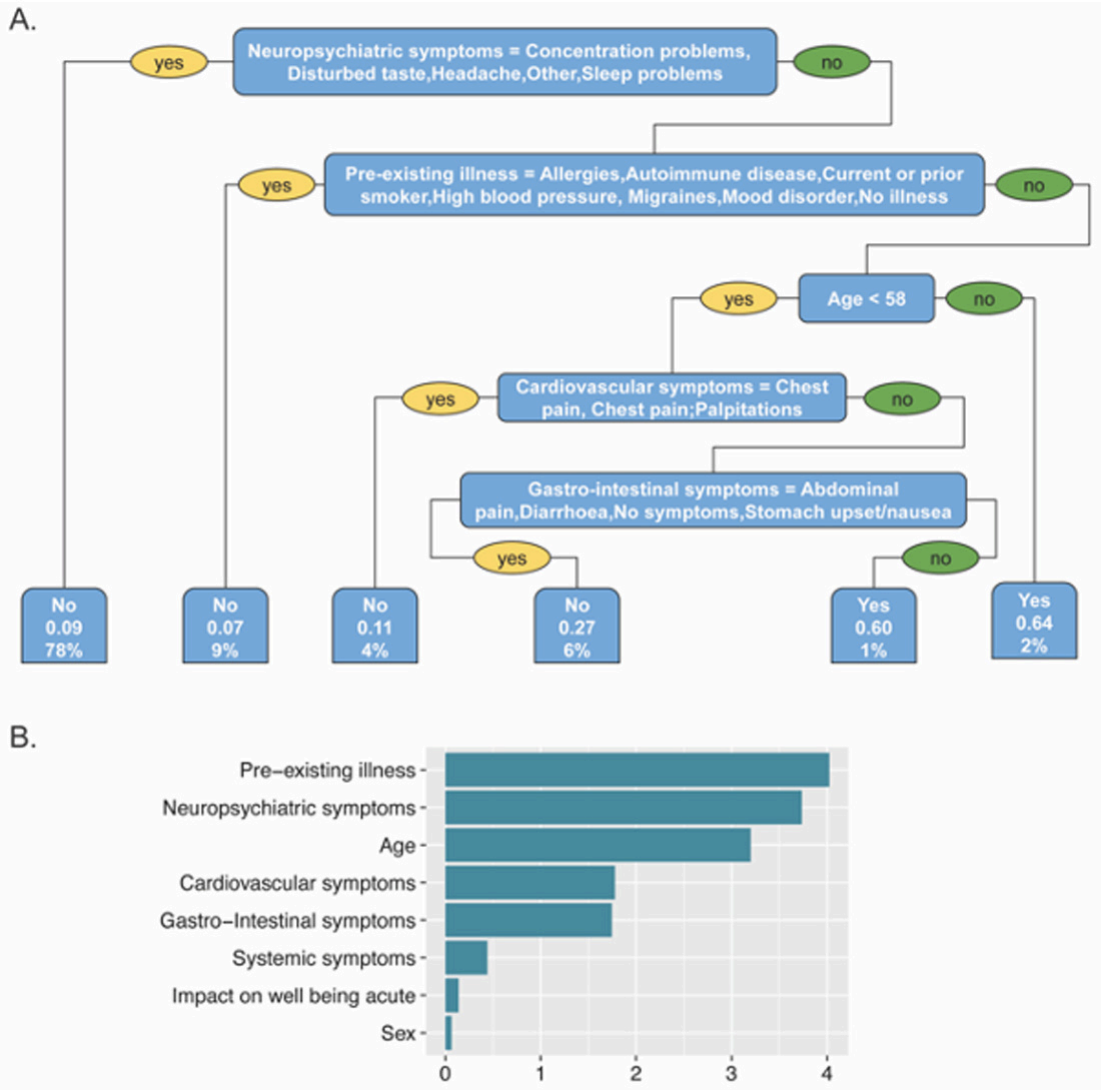
Prediction model for return to pre-COVID-19 level of health.

Interestingly, pre-existing comorbidities and neuropsychiatric symptoms during acute disease were the most important features of the model predicting a lack of return to pre-COVID level of health, which showed the highest predictive accuracy (86%) ( Figure 6).

### Fatigue, anxiety and depression

**Figure 9.**
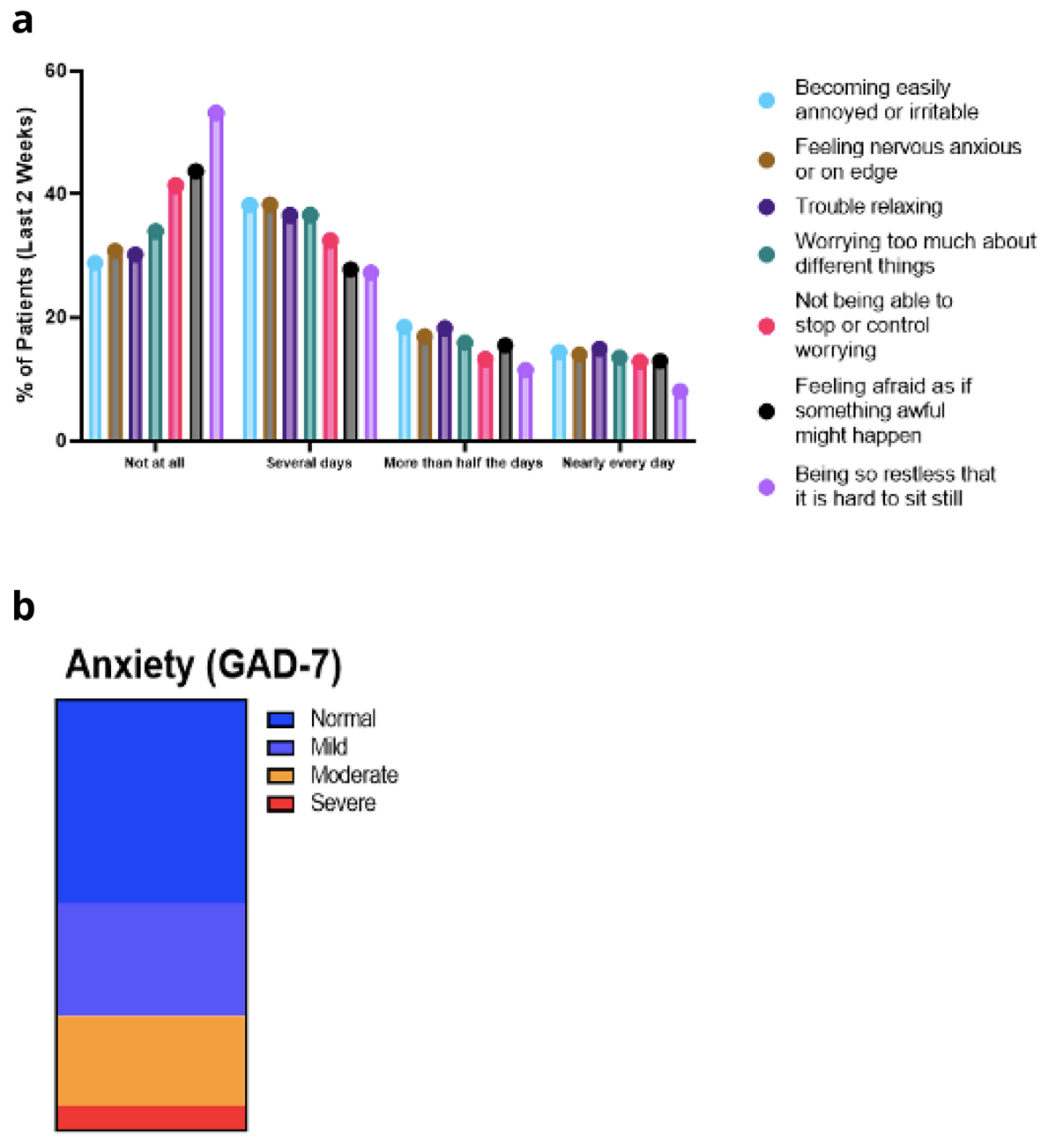
**a**. GAD-7 Symptoms. **b**. GAD-7 Anxiety Scale

***GAD-7.*** Disaggregated scores for GAD-7 are outlined in Figure 9a. Approximately 30% of patients were experiencing some level of anxiety for more than half the days of the previous two weeks ( Figure 9b). The only exception was the question relating to restlessness, with 20% of participants being so restless that it was hard to sit still for more than half the days of the previous two weeks. Overall, 34% of survey participants reported suffering an anxiety attack within the previous four weeks.

**Figure 10.**
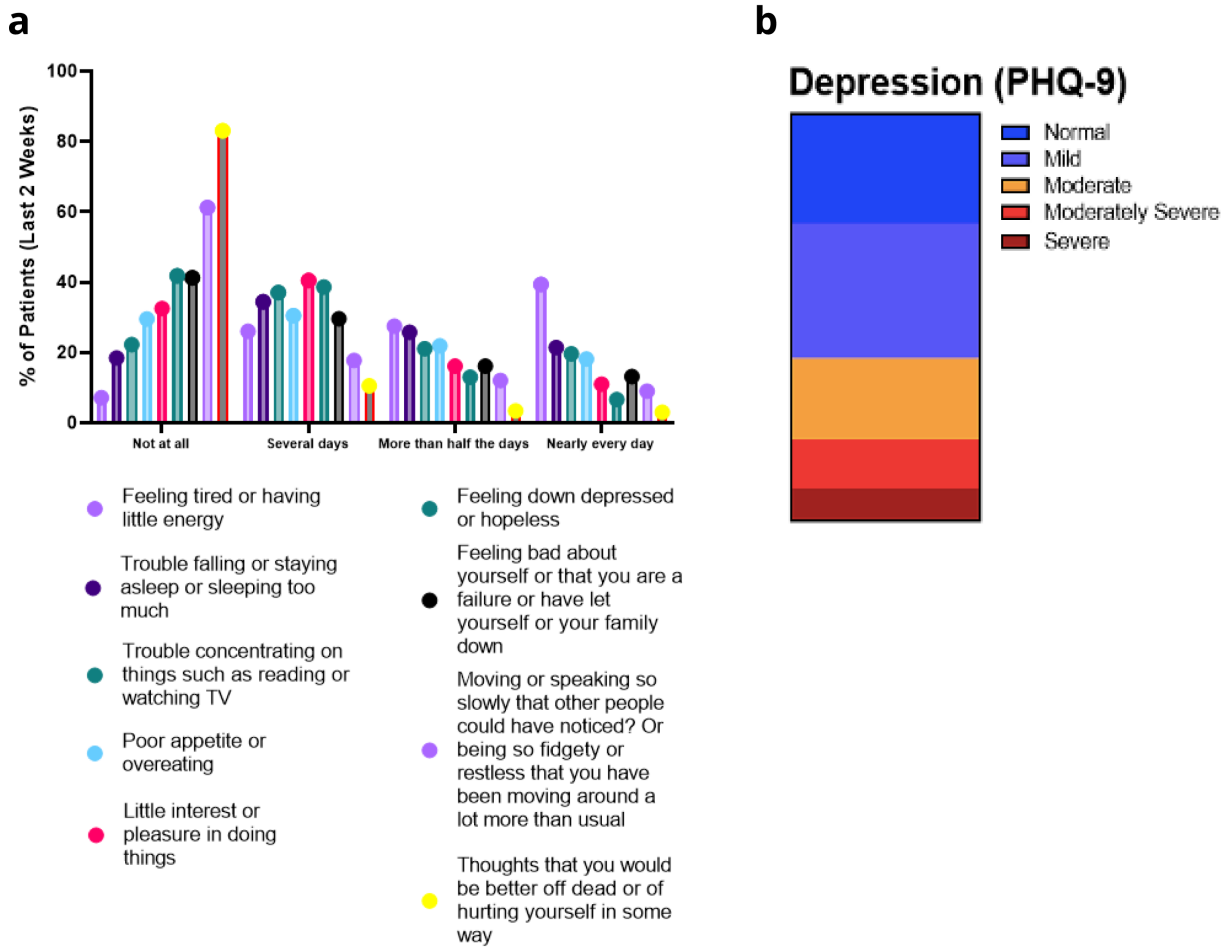
**a**. PHQ-9 Symptoms. **b**. PHQ-9 depression severity.

***PHQ-9.*** Disaggregated scores for PHQ-9 are outlined in Figure 10a. Again, the most frequent complaint was tiredness, or lack of energy, with many people experiencing disturbances of sleeping and eating patterns, accompanied by concentration difficulties, anxiety and little interest in doing things. Concerningly, 17% of patients had thoughts relating to self-harm or suicide at some stage during the previous two weeks ( Figure 10a). Using the PHQ-9 scale, 12% or 8% of respondents could be considered as moderately severe or severely depressed ( Figure 10b). Cluster analysis did not reveal any distinct clustering of symptoms associated with anxiety or depression.

**Figure 4.**
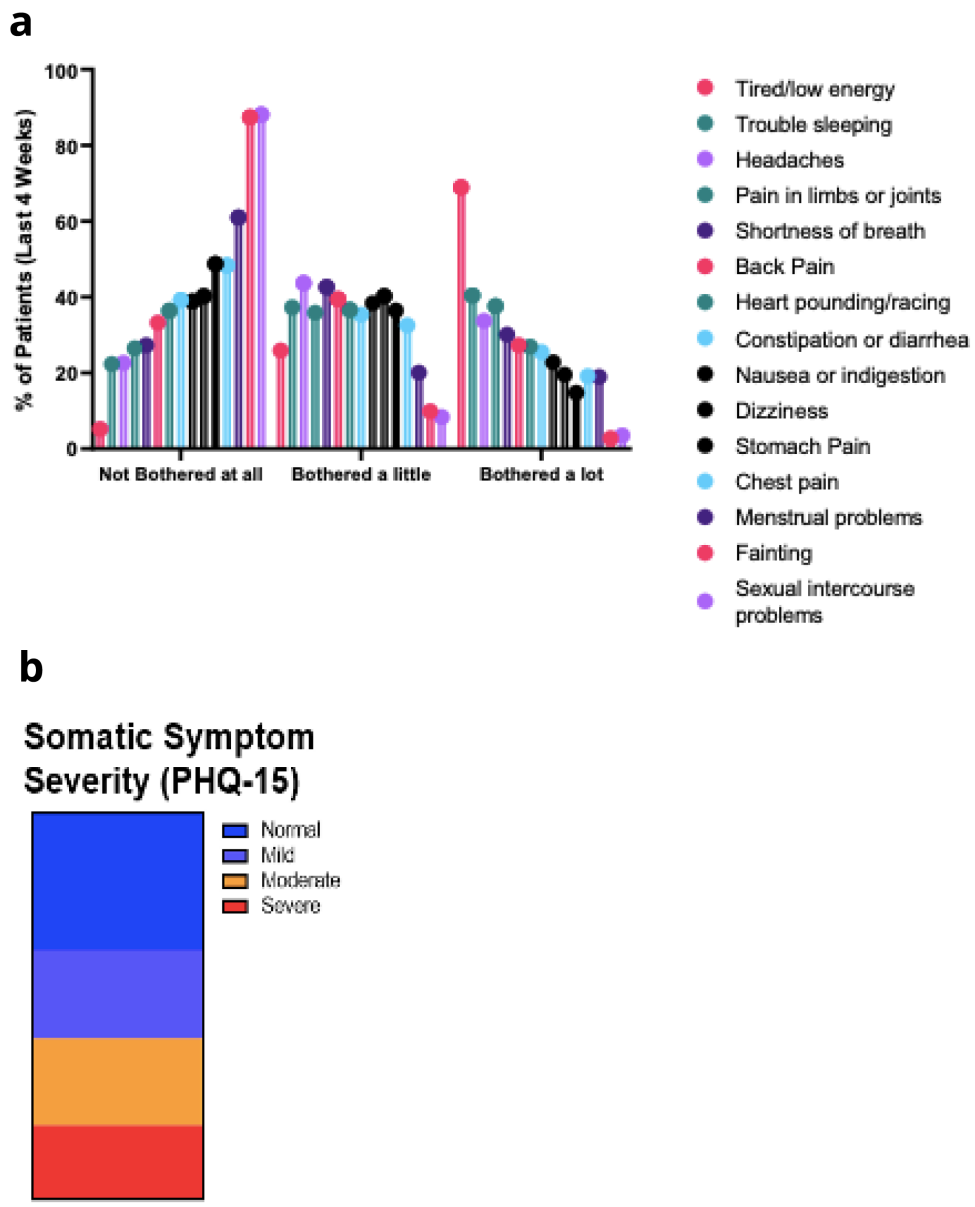
**a**. PHQ-15 symptoms. **b**. PHQ-15 Somatic Symptoms Severity.

***PHQ-15.*** Symptoms over the last four weeks were evaluated using the PHQ-15 and the symptoms that bothered them the most were being tired or low energy levels, trouble sleeping, headaches, pain in limbs or joints, and shortness of breath ( Figure 4a). Following scoring of the PHQ-15 responses, symptom severity could be considered moderate or severe for 30% or 25% of respondents respectively ( Figure 4b).

In response to questions on how these symptoms have impacted on your ability to do your work, take care of things at home, or get along with other people, participants rated their symptoms as not difficult at all (15.6%), somewhat difficult (47.4%), very difficult (21.5%) and extremely difficult (15.6%).

### Chalder fatigue scale

**Figure 7.**
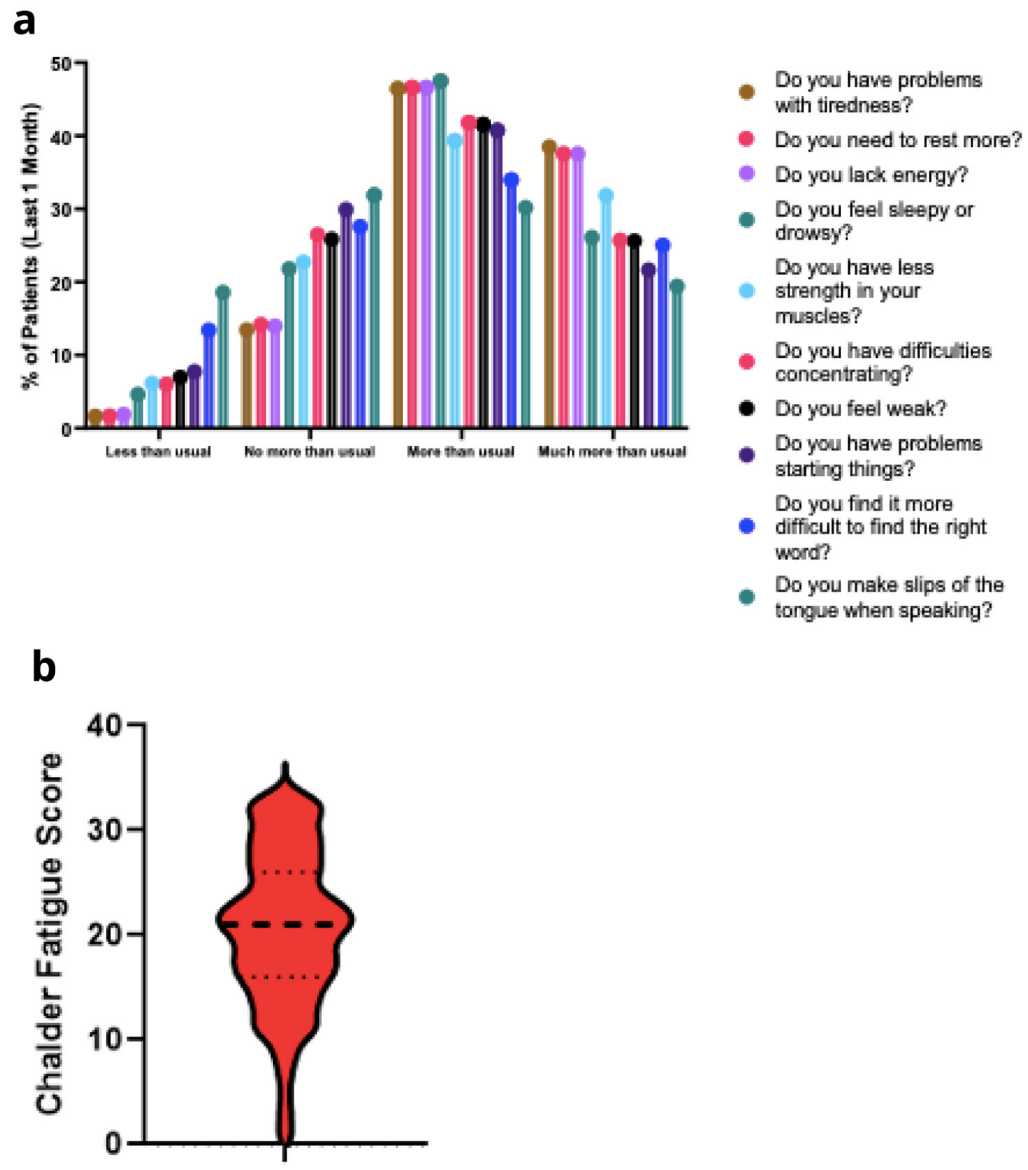
**a**. Chalder Fatigue Scale responses. **b**. Chalder Fatigue Scale score.

**Figure 8.**
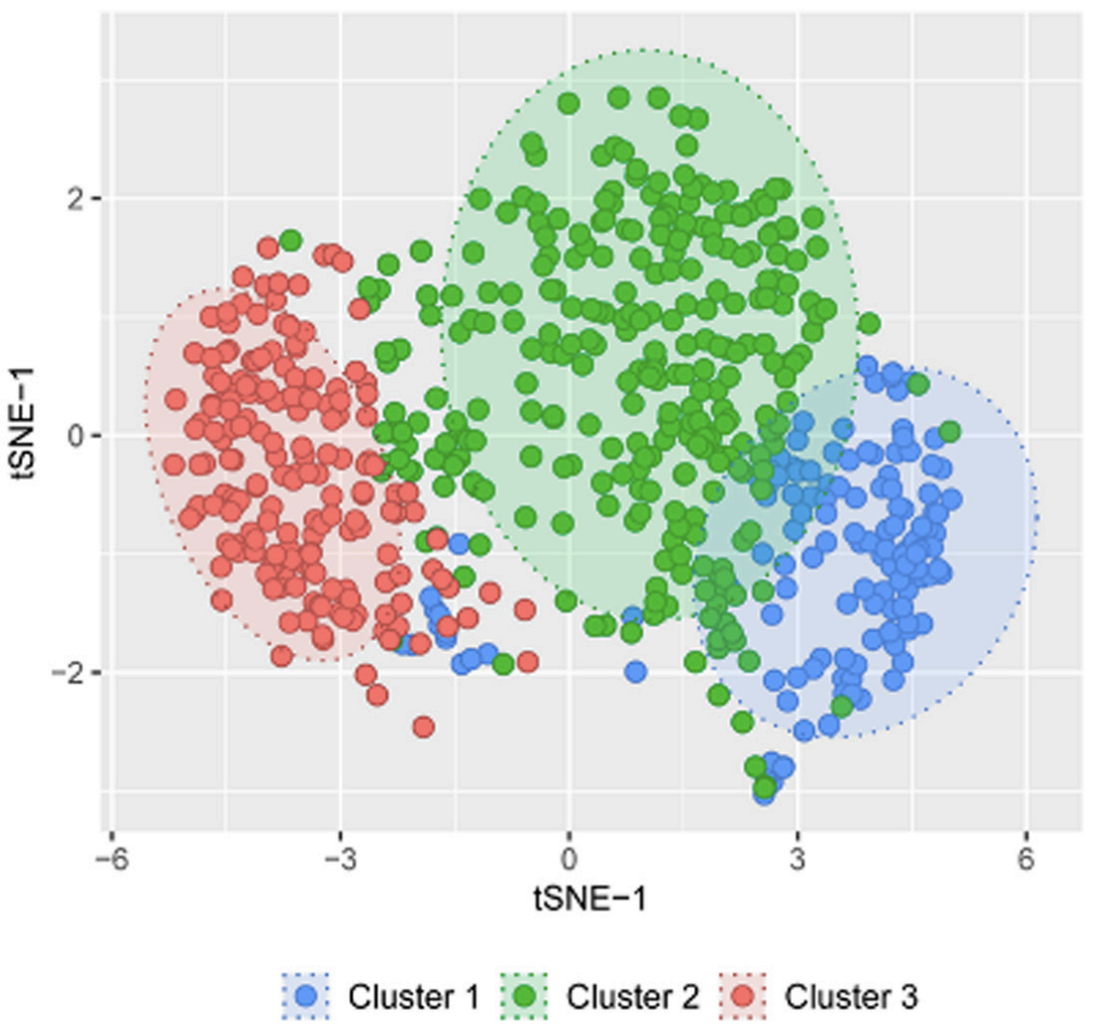
Cluster analysis of responses to Chalder Fatigue Scale questionnaire.

 In order to better characterise the physical and mental fatigue experienced by PASC patients, participants completed the Chalder fatigue scale (CFQ) as a component of the questionnaire. Within the last month, patients reported significant problems with feeling tired, weak, or lacking in energy ( Figure 7a). The mean CFQ score was 21.0 (SD 7.3) ( Figure 7b). Three distinct clusters were observed for patient responses to the Chalder fatigue questionnaire ( Figure 8). The specific questions that showed the best clustering were – Do you have problems with tiredness? Do you need to rest more? Do you lack energy? Do you have difficulties concentrating?

### Socioeconomic impacts

 Many of the survey participants have been restricted in their ability to work. 38% of participants rate the impact of their symptoms as severely limiting their ability to work, while there has been a moderate impact for a further 30% of participants. 60% of patients have been absent from work at some stage due to their PASC symptoms. 22% of participants have previously been in receipt of social welfare supports or pandemic unemployment payment (PUP) as a direct result of PASC, while 16% of participants are currently receiving supports due to their PASC illness. 

### Access to healthcare

52% of survey respondents strongly agree with the statement that accessing and navigating clinical support services for PASC has been difficult. In contrast, only 9% of participants strongly disagree with this statement. Within this survey cohort, 70% have seen their GP for symptoms of PASC, 30% have seen a hospital consultant and 24% have seen an occupational health physician while off work due to PASC. 31% of participants strongly agree or agree that their GP consultation was helpful, while 41% of participants strongly agree or agree that their consultant visit was helpful.

## Discussion

The results of this survey of an Irish cohort with PASC are in line with reports from other settings, and we confirm that patients with PASC reported prolonged, multisystem symptoms which can significantly impact quality of life, affect ability to work and cause significant disability.

The majority of respondents to our survey reported that their acute COVID-19 infection was detected by PCR (81%) or antigen testing (3%) however 16% did not have a COVID-19 diagnosis confirmed. It is widely accepted that the number of SARS-CoV-2 infections reported at a population level is an underestimate. The Study to Investigate COVID-19 Infection in People Living in Ireland (SCOPI) surveillance study examined seroprevalence of SARS-CoV-2 in the Irish population following the first wave of COVID-19 and estimated that up to three times the number of reported cases had been infected at that time ^30^.

PASC has been reported following severe, mild, or even asymptomatic SARS-CoV-2 infection ^31^. In our survey 62% of participants reported mild or moderate acute COVID-19 infection with 9% requiring hospital assessment. Development of potentially long-term debilitating symptoms following mild infection is concerning from a public health perspective given overall numbers in the population that have been and continue to be infected.

Large studies have consistently demonstrated that women are less likely than men to experience severe disease or death from COVID-19 ^32^, however they are at higher risk of developing PASC and they are over represented in published studies on PASC ^33– 35^. This finding is reflected in our survey where 88% of respondents were female. The reason that PASC predominantly affects women is not yet understood however it is not a new phenomenon. Women are known to be up to four times more likely to get myalgic encephalomyelitis, or chronic fatigue syndrome (ME/CFS), a condition thought to have infectious origins in the majority of cases ^36^. Additionally, women are also more likely to experience symptoms of depression ^37^.

Many PASC patients will experience improvement or resolution of symptoms over time however there is a proportion in whom symptoms persist in the longer term. Almost 90% of participants in our survey reported ongoing symptoms at time of completion of the survey. The median number of months participants reported symptoms for was 12 or more highlighting the enduring nature of symptoms for some.

Over 200 symptoms have been reported to be associated with PASC ^38^ and reported symptoms frequently affect multiple organ systems. More than two thirds of PASC patients in our survey reported fatigue, post-exertional malaise, palpitations, chest pain, stomach upset/nausea, memory problems, muscle pain and joint pain. These symptoms are consistent with common symptoms reported in other studies. Additionally, most patients reported symptoms affecting multiple systems with a median of 8 PASC symptoms reported.

The impact of COVID-19 on health-related quality of life is significant in both acute COVID and PASC patients ^39^. A high proportion of PASC patients in our study indicated moderate or severe limitation in their ability to carry out their usual activities (48%), and in their mobility (27%), with 44% experiencing a moderate, or higher, level of pain and 33% reporting a moderate, or higher, level of anxiety or depression. Overall participants reported that disease symptoms had moderate (43%) or severe impact (33%) on general well-being.

The COVID-19 pandemic and associated restrictions have had a significant impact on mental health. In Ireland studies have reported symptoms of anxiety and depression in 20–28% of the general population with up to 4% experiencing thoughts of self-harm or suicide ^40, 41^. Findings from the Healthy Ireland 2018 survey, a national representative survey, reported rates of 6% of self-reported depression and anxiety before the COVID-19 pandemic ^42^.

Sars-CoV-2 infection has been associated with a wide range of neuropsychiatric symptoms in both the acute and convalescent stages ^43^. In our survey, 29% – 45% of participants reported moderate levels of anxiety and depression. Concerningly, 17% reported experienced thoughts of self-harm or suicide. Survey participants also reported high levels of physical symptoms using the PHQ-15 somatic screening tool as part of the PHQ-SADS. It is important to highlight that while aetiology of many symptoms associated with PASC are currently unknown, a number of mechanisms have been identified and are being actively researched ^7– 13^. Given the identified prevalence of incident mental health disorders in patients with PASC, focused screening for mental health disorders in addition to physical illness supports should be made available to patients with PASC in Ireland. The evidence to date suggests there has not been an objective rise in suicidal behaviour or completed suicide ^44^.

Our finding that neuropsychiatric symptoms during PASC can be predicted in part by respiratory and gastrointestinal symptoms during acute disease is novel, and suggests a mechanism of action that might involve modulation of immune and metabolic processes at these mucosal sites, as we have previously described ^12, 13^. This also suggests that efforts to treat PASC neuropsychiatric symptoms might require multidisciplinary approaches that include respiratory, gastrointestinal, immunological and microbiome interventions.

45% of respondents to our survey reported memory problems. Many post COVID patients describe persistent ‘brain fog’ after resolution of acute infection. Zhao *et al.* measured cognitive functional parameters in subjects who had COVID compared with those who had not ^45^. Compared with the control group, subjects who had previous COVID demonstrated worse episodic memory and concentration. Importantly, none of the subjects in this study had a diagnosis of PASC or were seeking care in specialist clinics. This suggests that ‘brain fog’ may be sub-clinical and may occur in the absence of other PASC symptoms.

In addition to the adverse physical and psychological outcomes of COVID-19, many patients suffer additional financial losses due to missed work days. In our study, almost two thirds of respondents missed work days due to ongoing symptoms. In a similar survey by Davis *et al.*, 45% of respondents required a reduced work schedule and 22% were no longer working due to illness ^35^.

In a Swiss study of recovered COVID patients, the researchers found that persistent fatigue, dyspnoea and depression were more likely to require further healthcare contacts ^46^. Similarly, most of respondents to our survey indicated previous visits to their GP, with some patients needing further evaluation in hospital.

Our study has a number of limitations. Survey respondents self-selected to participate in the questionnaire. Respondents were predominantly female, white and with a high level of education thus results may not be generalisable to the broader population. Symptoms were self-reported and may be confounded by reporter bias. There was no control group to compare response outcomes, although there is published data looking at anxiety and depression in the general Irish population available during the pandemic period. Screening tools used in the survey are not diagnostic and are not a substitute for clinical diagnostic interview and exam by physicians and mental health professionals. We did not capture variant of infection or vaccination status at time of acute COVID-infection which may also have been factors in severity of acute illness and persistence of residual symptoms.

Our survey demonstrates that patients with PASC represent a significant burden on both primary and secondary care. Moreover, the results of our survey show that over half of subjects found it difficult to access support services. Our findings indicate that there is a need for specialist, multi-disciplinary approach to post COVID care pathways.

Given the diversity of multisystem symptoms, treatment of patients with PASC requires a multidisciplinary cross specialty approach including evaluation, symptomatic treatment, and treatment of underlying problems, physiotherapy, occupational therapy and mental health supports ^47^.

As we enter a new phase of the pandemic with the majority of our population vaccinated a greater focus needs to be placed on addressing PASC and other long-term impacts of COVID-19 infection. With proper research investment and collaborative efforts with patients, we can find ways to improve outcomes for patients suffering from PASC.

## Data availability

### Underlying data

figshare: Long COVID Survey Data. https://doi.org/10.6084/m9.figshare.19364828.v1 ^48^

### Extended data

Figshare: Long COVID Survey. https://doi.org/10.6084/m9.figshare.19447919 ^23^

Data are available under the terms of the Creative Commons Attribution 4.0 International license (CC-BY 4.0).
