# Supplementary material for: Impact of Long COVID on health and quality of life
Source: HRB Open Res. 2022 Apr 22;5:31. [Version 1] doi: 10.12688/hrbopenres.13516.1 (PMC9440374; doi:10.12688/hrbopenres.13516.1)
Supplement: Peer review history

# Peer review: Reviewer response for version 1

**DOI:** 10.21956/hrbopenres.14747.r32610

**Published:** 2022-09-02

**Authors:** Jennifer L. P. Protudjer

**Affiliations:** 1 The Children’s Hospital Research Institute of Manitoba, Winnipeg, MB, Canada

Thank you for the opportunity to review the paper titled, “Impact of Long COVID on health and quality of life” written by O’Mahony and colleagues. In this paper, the authors aimed “to measure the impact of post-acute sequelae of COVID-19 on quality of life, mental health, ability to work and return to baseline health within the Irish population.” This is an interesting and well-written paper that provides insight into the burdens of Long COVID, and makes some important calls for action. Some comments for consideration are listed below.

1. Methods: It would be helpful to explicitly state why data collection began on 28 September 2021. Although this is implied with the mention of the WHO’s clinical case definition, developed in October 2021, please explicitly indicate if this was the reason. If not, explain the rationale for selecting this date.
2. Methods: Data collection occurred up to 30 days to allow participants to return to the survey at a later time. While reasonable to allow participants time to complete the extensive questionnaire package, it is possible that this also contributed to biased answers. Participants’ experiences of long COVID symptoms and their perceptions of these symptoms may change over the 30 days.
3. Results: If participants did not have to complete a question before proceeding to the next, how many missing data points were in the dataset? If missing data were simply excluded, it is necessary to consider how this might affect the analyses and the interpretation.
4. Results: It is unclear if differences were considered in consideration to duration of long COVID. While the minimum number of symptomatic days was 14, it is unclear if and how the duration of Long COVID differentially predicted the outcomes.
5. Results: Were there any differences in outcomes between those with self-reported confirmed vs. suspected COVID-19 with symptoms lasting over 14 days?
6. Results, PHQ-9: The authors wrote “Concerningly, 17% of patients had thoughts relating to self-harm or suicide at some stage during the previous two weeks” This is an incredibly concerning finding, and thus should be positioned elsewhere, rather than in the middle of a paragraph.

Is the work clearly and accurately presented and does it cite the current literature?

Yes

If applicable, is the statistical analysis and its interpretation appropriate?

Partly

Are all the source data underlying the results available to ensure full reproducibility?

Yes

Is the study design appropriate and is the work technically sound?

Yes

Are the conclusions drawn adequately supported by the results?

Yes

Are sufficient details of methods and analysis provided to allow replication by others?

Yes

Reviewer Expertise:

allergy, epidemiology, mixed methods.

I confirm that I have read this submission and believe that I have an appropriate level of expertise to confirm that it is of an acceptable scientific standard.

# Peer review: Reviewer response for version 1

**DOI:** 10.21956/hrbopenres.14747.r32609

**Published:** 2022-08-16

**Authors:** Joanna S. Makowska

**Affiliations:** 1 Department of Rheumatology, Medical University of Lodz, Łódź, Poland

The reviewed manuscript is a questionnaire-based study analyzing the prevalence and impact of PASC on health and quality of life among responders from 31 countries. Presented study is using validated tools to assess patients’ health, anxiety, fatigue, and depressive symptoms. It also contained 30 questions including demographic data, comorbidities, COVID-19 severity, etc.

 The authors identified limitations of the study, the main one is the lack of a control group, which is also the problem in other studies concerning PASC. Secondly, the study is based on self-administered questionnaires, and also the data on positive results of COVID confirmation is based on PCR or antibody tests results reported by the patients- which is a weak point of the study, as various methods were used to confirm infection.  

**Major comments.**

1. What was the method of confirmation of COVID-19 infection - was it PCR results, antigenic tests, or were antibodies also assessed- as it was written in the Method section? Could the Authors provide the questions posed in the questionnaire? 
2. According to the data presented, 84% of patients had confirmed COVID-19 infection while in 16% of the patients the diagnosis was made based on patients' beliefs- the group was analyzed together.  It would be very interesting to look if those groups of patients differ in terms of the prevalence of certain symptoms. In ex. there is a very interesting study from France ^1^ showing that patients without COVID -19 infection (negative serology for COVID-19 infection) but being sure that they had infection also presented very often PASC syndrome- which suggests that maybe some symptoms of PASC can be attributed, in at least some patient, not to real viral infection but anxiety, depression, and stress caused by pandemic, lockdown. 
3. Did the Authors look for the association between preexisting comorbidities and the risk of PASC? For ex. are patients with diagnosed depression more prone to develop PASC?
4. In the results section Authors described that pre-existing comorbidities are predicting a lack of a pre-COVID level of health, could the Authors specify which comorbidities?
5. Did the Authors include the question about previous vaccination against COVID-19 in the questionnaire? If yes, did the vaccination influence the prevalence of PASC?

Is the work clearly and accurately presented and does it cite the current literature?

Yes

If applicable, is the statistical analysis and its interpretation appropriate?

Yes

Are all the source data underlying the results available to ensure full reproducibility?

No source data required

Is the study design appropriate and is the work technically sound?

Yes

Are the conclusions drawn adequately supported by the results?

Yes

Are sufficient details of methods and analysis provided to allow replication by others?

Yes

Reviewer Expertise:

immunology, epidemiology,

I confirm that I have read this submission and believe that I have an appropriate level of expertise to confirm that it is of an acceptable scientific standard.

## References

[1] : Association of Self-reported COVID-19 Infection and SARS-CoV-2 Serology Test Results With Persistent Physical Symptoms Among French Adults During the COVID-19 Pandemic. *JAMA Internal Medicine* .2022;182(1) : 10.1001/jamainternmed.2021.6454 10.1001/jamainternmed.2021.6454 34747982PMC8576624
